# Healthcare Providers’ and Pregnant People’s Preferences for a Preventive to Protect Infants from Serious Illness Due to Respiratory Syncytial Virus †

**DOI:** 10.3390/vaccines12050560

**Published:** 2024-05-20

**Authors:** Kathleen M. Beusterien, Amy W. Law, Martine C. Maculaitis, Oliver Will, Lewis Kopenhafer, Patrick Olsen, Brett Hauber, Jeffrey T. Vietri, Joseph C. Cappelleri, Joshua R. Coulter, Kimberly M. Shea

**Affiliations:** 1 Oracle Life Sciences, Austin, TX 78741, USA; 2 Pfizer Inc., New York, NY 10001, USA; 3 Pfizer Inc., Collegeville, PA 19426, USA; 4 Pfizer Inc., Groton, CT 06340, USA

**Keywords:** healthcare providers, maternal vaccination, monoclonal antibodies, pregnant people, respiratory syncytial virus, stakeholder preferences

## Abstract

We assessed the impact of respiratory syncytial virus (RSV) preventive characteristics on the intentions of pregnant people and healthcare providers (HCPs) to protect infants with a maternal vaccine or monoclonal antibodies (mAbs). Pregnant people and HCPs who treated pregnant people and/or infants were recruited via convenience sample from a general research panel to complete a cross-sectional, web-based survey, including a discrete choice experiment (DCE) wherein respondents chose between hypothetical RSV preventive profiles varying on five attributes (effectiveness, preventive type [maternal vaccine vs. mAb], injection recipient/timing, type of medical visit required to receive the injection, and duration of protection during RSV season) and a no-preventive option. A best–worst scaling (BWS) exercise was included to explore the impact of additional attributes on preventive preferences. Data were collected between October and November 2022. Attribute-level preference weights and relative importance (RI) were estimated. Overall, 992 pregnant people and 310 HCPs participated. A preventive (vs. none) was chosen 89.2% (pregnant people) and 96.0% (HCPs) of the time (DCE). Effectiveness was most important to preventive choice for pregnant people (RI = 48.0%) and HCPs (RI = 41.7%); all else equal, pregnant people (RI = 5.5%) and HCPs (RI = 7.2%) preferred the maternal vaccine over mAbs, although preventive type had limited influence on choice. Longer protection, protection starting at birth or the beginning of RSV season, and use for both pre-term and full-term babies were ranked highest in importance (BWS). Pregnant people and HCPs strongly preferred a preventive to protect infants against RSV (vs. none), underscoring the need to incorporate RSV preventives into routine care.

## 1. Introduction

Respiratory syncytial virus (RSV) is a common viral infection that often causes mild cold-like symptoms but may result in serious disease in children or older adults. It is a major cause of acute lower respiratory infections (e.g., bronchiolitis and pneumonia) in infants and a leading contributor to infant hospitalizations; it has been estimated that 1–2% of children < 6 months old may require hospitalization due to RSV [1]. Each year in the United States (US), RSV leads to, on average, approximately 58,000 hospitalizations with 100–500 deaths among children <5 years old [2]. Many children are infected by RSV in their first year of life, and by age 2, infection is nearly universal. Most infections result in mild disease, but infections can be severe in very young infants (i.e., <6 months of age) and in infants with other risk factors, such as pre-term birth [1]. A systematic literature review reported that, in addition to the risk of mortality, RSV in infants is associated with various long-term health complications, such as recurrent wheezing and asthma, or impaired lung function [3].

Current treatment for RSV disease in infants typically involves supportive care, and preventives are limited. The monoclonal antibody (mAb) palivizumab was approved by the US Food and Drug Administration (FDA) in 1998 and is administered via monthly injections to the infant during the RSV season. In 2014, the American Academy of Pediatrics recommended that palivizumab be restricted to high-risk infants—principally pre-term infants (≤35 weeks gestational age) and/or those with respiratory or cardiac conditions [4]. Palivizumab has been found to be effective in reducing RSV hospitalization in pre-term infants, children with bronchopulmonary dysplasia, and infants with hemodynamically significant congenital heart disease, as well as in reducing recurrent wheezing episodes after RSV hospitalization [5]. However, palivizumab’s monthly injection requirements, along with the costs of therapy, may limit the proportion of patients who receive it.

Two approaches for RSV prevention have recently been developed. One infant RSV prevention strategy that was recently approved by the FDA is maternal vaccination, which is currently indicated for use during the third trimester of pregnancy (gestational age of 32 to 36 weeks) [6]. Presently, there is only one available FDA-approved maternal vaccine option; it is recommended to be administered as a single dose to pregnant people between September and January in most places in the continental US to ensure protection is conferred to the infant during the RSV season [7]. Vaccinating pregnant persons against RSV results in the transfer of vaccine-induced maternal antibodies across the placenta, conferring immunity to infants against RSV starting at the time of birth [8]. Another recently developed immunization approach for RSV prevention is the administration of long-acting mAbs to infants early in life [9], such as nirsevimab, which was approved by the FDA in July 2023 [10]. Long-acting mAbs, which are indicated for all infants, have been engineered to have a longer half-life than palivizumab, thus removing the need for repeated administration to cover the time of highest RSV risk. Specifically, nirsevimab is recommended to be administered as a single dose, with administration timed to coincide with the period shortly before or during the RSV season [11]. Presently, there is no preferential recommendation for RSV immunization options, as the administration of either the maternal vaccine or long-acting mAb has been shown to effectively protect infants from RSV-related lower respiratory tract infections. However, in light of a supply shortage during the 2023–2024 RSV season, the Centers for Disease Control and Prevention (CDC) recommended that available doses of nirsevimab be reserved for infants at highest risk for severe illness from RSV [12]. To the extent that maintaining adequate supply remains a challenge, nirsevimab may only play a limited role in protecting the broader population of infants against RSV, highlighting the need for readily available alternative options.

Given the evolving RSV immunization landscape, it is essential to elicit the perspectives of key stakeholders to inform public health strategies to prevent RSV in infants. Accordingly, this study aimed to understand the views of healthcare providers (HCPs) who provide prenatal or infant care, including maternal or infant immunizations, and those of pregnant people regarding hypothetical maternal vaccine and long-acting mAb options to protect infants against serious illness from RSV. To our knowledge, this research is the first to quantify the extent to which HCPs and pregnant people would choose to use RSV preventive products, or forgo a preventive, and to evaluate their perceptions about the importance of multiple attributes (e.g., effectiveness, preventive type, etc.) that may influence their choice of RSV preventive.

## 2. Materials and Methods

### 2.1. Study Design

This cross-sectional, non-interventional study incorporated a discrete choice experiment (DCE) and a best–worst scaling (BWS) exercise to assess the willingness to use a preventive product to protect an infant against serious illness from RSV, and to identify the key drivers guiding the decision to use a preventive product [13,14]. Cognitive pre-test interviews with 8 HCPs and 8 pregnant people were conducted prior to fielding the survey to ensure the survey instrument was medically relevant, concise, and viewed as being manageable by respondents.

All respondents provided informed consent electronically, and no personally identifying data were collected. All participants who completed the survey received fair market value compensation for their time.

### 2.2. Study Population

Participants were recruited through Kantar’s LifePoints panel, a general research panel with over 20 million panel members in the US across all four geographic areas: Northeast (14%), Midwest (21%), South (44%), and West (21%). Potential panelists are asked to complete an in-depth registration profile, which includes numerous logic checks to ensure respondent quality, and a double opt-in process. Panelists receive points for completing a survey, which are deposited into their accounts and can later be redeemed for products, online gift certificates, or cash remuneration. All potential participants were invited to take part in the study via email; they first accessed the study screener to confirm their eligibility. Prior to proceeding to the main survey, eligible participants were provided with an electronic informed consent form and asked to confirm their informed consent before proceeding to the main survey. The data were collected between October and November 2022, using a web-based survey of HCPs and adult pregnant people in the US. To achieve sufficiently precise preference estimates for the DCE and BWS, the target sample sizes for this study were approximately 300 for HCPs who provide care to infants or pregnant people and approximately 1000 for pregnant people.

To determine sample sizes for aggregate level full-profile DCE modelling, we used the formula *nta*/*c* > 1000, where *n* is the number of respondents, *t* is the number of choice tasks, *a* is the number of alternative profiles presented in each task, and *c* is equal to the largest number of levels for any one attribute [14]. With target sample sizes of 300 HCPs and 1000 pregnant people, 12 choice tasks, two alternative RSV preventive profiles presented in each choice task, and a maximum of four levels per attribute, the formula result is 1800 and 6000 for HCPs and pregnant people, respectively, both of which are >1000, indicating that we have a sufficient sample size to obtain relatively precise utility estimates from the analysis of the DCE data for both cohorts. To compute the needed sample size for BWS, we used binomial sampling as an approximation, with the goal of finding a sample size that would estimate the rate of selecting an item as best to within ±4%. From binomial sampling theory, the item would need to be seen 600 times. Since an item is seen 5.14 times by a respondent in a 14-item approximate balanced inventory block design, the binomial approximation would need at least 117 (=600/5.14) respondents to ensure sufficiently precise estimates from the analysis of BWS data [13].

To capture the perspectives of different clinicians involved in making preventive recommendations, the recruiting plan aimed to collect data from an approximately equal number of HCPs that provide care to infants and pregnant people, with roughly 100 HCPs from each of the following specialties: pediatricians, obstetricians (OBs)/obstetrician-gynecologists (OB/Gyns), and other HCP types (nurse practitioners, nurse midwives, and family practitioners). HCPs were eligible to participate in the survey if they met all the following inclusion criteria: specialized in obstetrics/gynecology, pediatrics, primary care, internal medicine, or midwifery; had provided prenatal care to pregnant persons or healthcare to infants aged ≤6 months in the past 2 months; had ≥2 years of experience treating pregnant people or infants aged ≤6 months; had prescribed, recommended, or administered an immunization to a pregnant person or infant aged ≤6 months in the past 6 months; had a current, active, unrestricted license to practice in the US; and were able to answer questions in English. HCPs who reported an affiliation with the study sponsor, either directly or indirectly through a family member, or were unwilling or unable to provide informed consent, were excluded.

To ensure the pregnant person sample reflected the diversity of the US pregnant population, the recruiting plan targeted a minimum of 400 participants pregnant for their first birth and 400 individuals expecting their second or later birth, and at least 200 pregnant people from each of the following racial/ethnic groups: White, Black/African American, and Hispanic/Latina. Pregnant people were included in the study based on all the following criteria: age ≥ 18 years; current pregnancy at the time of the study; residence in the US; and ability to answer questions in English. Pregnant people who were unwilling or unable to provide informed consent or information regarding the number of previous births were excluded from the study.

### 2.3. Measures

All participants in this study completed both the DCE and the BWS choice tasks. In the DCE exercise, each respondent saw 12 choice tasks in which each RSV preventive option was defined by a combination of levels of the five attributes included. Participants chose between two hypothetical preventive product profiles and an opt-out ‘No Preventive Product’ alternative in each choice task. In the BWS exercise, each respondent saw 18 choice tasks displaying a set of 4 attributes at a time, with a total of 14 attributes assessed. Participants were asked to choose the best and the worst attributes among those displayed in each BWS task. A brief overview of the DCE and BWS methodologies is presented below.

**Figure 1:**
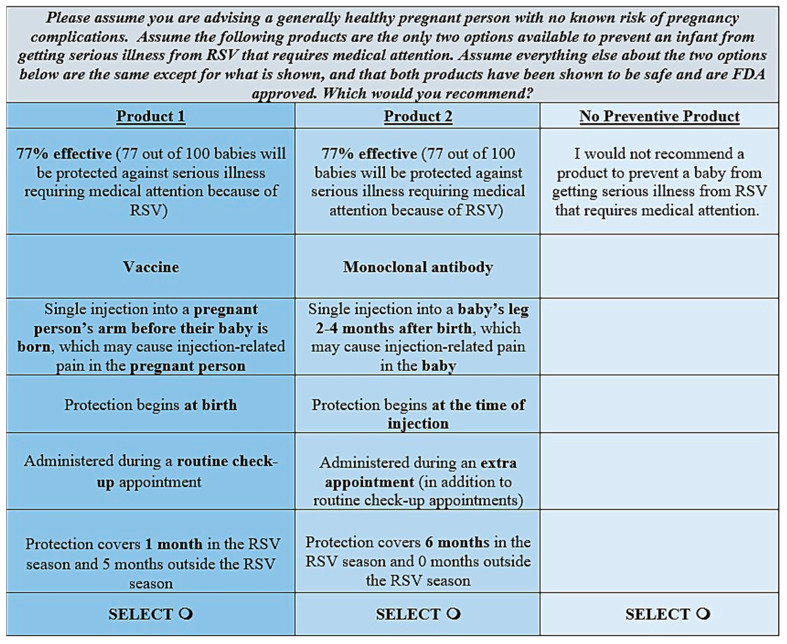
Example Discrete Choice Experiment Choice Task. Note. Injection recipient and timing of protection were presented as separate attributes for ease of comprehension, but the attributes were linked in how they were presented to preclude illogical combinations (e.g., “Protection begins at birth” never appeared with “Single injection into a baby’s leg 2–4 months after birth”); they were treated as one attribute in analyses. The same attributes and levels were included in the pregnant person survey, except the term “pregnant woman” was used instead of “pregnant person” based on feedback received in pilot interviews. The DCE question in the pregnant person survey was: “Please imagine that a healthcare provider has suggested one of the following options to help prevent your baby from getting serious illness from RSV that requires medical attention. Assume there are only two preventive products available. Assume everything else about the two products below are the same except for what is shown, and that both products have been shown to be safe and are FDA approved”. Abbreviations. FDA: Food and Drug Administration; RSV: respiratory syncytial virus.

The DCE was included in the survey to assess potential trade-offs among RSV preventive attributes and the willingness of respondents to use or recommend an RSV preventive similar to those recently approved or in late-stage development. The DCE is rooted in economic theory and is based on the principle that product alternatives can be decomposed into attributes of interest [15]. The attractiveness of a product to individuals depends on their relative preferences for these attributes, which is expressed by the frequency with which they choose product profiles with the preferred features. The attributes and levels included in the DCE, as well as an example DCE choice task, are presented in Table 1 and Figure 1, respectively. Attributes and levels in the DCE were informed by the available literature; the sources for the attribute levels are provided in Table 1.

The levels for each attribute in the present study represent the range observed or expected to be observed in clinical trials or real-world studies of RSV preventive products that were available at the time of the study or were expected to become available in the foreseeable future. The combinations of levels defining each preventive product profile in the DCE, as well as the pairing of profiles in each choice task, were based on a balanced overlap design, which optimizes overall efficiency in terms of balance (each level was shown approximately an equal number of times), minimal overlap (repeating levels within the same task), and orthogonality (levels may be evaluated independently of other levels). All respondents answered a different set of choice tasks [16].

**Table 1:** Discrete Choice Experiment Attributes and Levels.

Attribute	Description	Levels
Effectiveness [17,18,19]	[INSERT LEVEL] effective. ([INSERT LEVEL] out of 100 babies will be protected against serious illness requiring medical attention because of RSV)	50%, 50 63%, 63 77%, 77 90%, 90
Type of prevention [20]		Vaccine Monoclonal antibody
Recipient of injection and timing of protection [9,17]	Split into two rows in tasks. Single injection into [INSERT LEVEL], which may cause injection-related pain in the [INSERT LEVEL]. Protection begins [INSERT LEVEL]	Single injection into a pregnant woman’s (pregnant person’s) arm before their baby is born, which may cause injection-related pain in the woman (pregnant person). Protection begins at birth Single injection into a baby’s leg within 14 days after birth, which may cause injection-related pain in the baby. Protection begins at the time of injection Single injection into a baby’s leg 2–4 months after birth, which may cause injection-related pain in the baby. Protection begins at the time of injection
Type of visit required [20]	Administered during [INSERT LEVEL]	a routine check-up appointment an extra appointment (in addition to routine check-up appointments)
Protection corresponds to season (RSV is a risk for 6 months) [9,18,21,22]	Protection covers [INSERT LEVEL]	1 month in the RSV season and 5 months outside the RSV season 3 months in the RSV season and 3 months outside the RSV season 4.5 months in the RSV season and 1.5 months outside RSV season 6 months in the RSV season and 0 months outside the RSV season

Note. The same attributes and levels were included in the DCEs for HCPs and pregnant people, except the term “pregnant person” was used in the DCE for HCPs, and “pregnant woman” or “woman” was used in the DCE for pregnant people. Abbreviations. RSV: respiratory syncytial virus.

**Figure A1:**
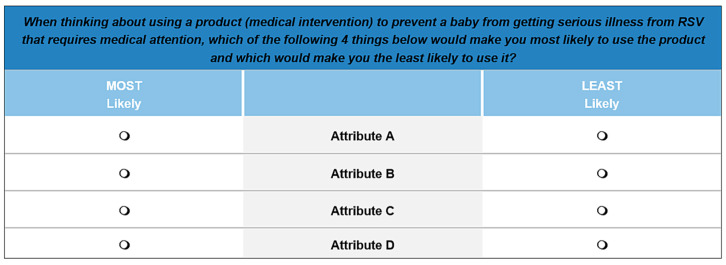
Example Best–Worst Scaling Choice Task. Note. The corresponding BWS question in the HCP survey was “When thinking about using a product (medical intervention) to prevent a baby from getting serious illness from RSV that requires medical attention, which of the following 4 things below would make you most likely to use the product and which would make you the least likely to use it?” Abbreviations. RSV: respiratory syncytial virus.

The BWS exercise was included to understand how HCPs and pregnant people prioritize a broader set of RSV preventive product attributes than could be accommodated in the DCE [13,23]. BWS yields a rank ordering of the relative importance (RI) of selected product features or attributes. Specifically, in the current study, we used the BWS object case (i.e., case 1) methodology, which allows for the perceived importance of each attribute that contributes to an individual’s RSV preventive choice to be evaluated relative to the perceived importance of all other attributes. The attributes included in the BWS exercise, as well as an example BWS choice task, are presented in Table 2 and Figure A1, respectively.

In addition, data on sociodemographic characteristics (e.g., age, race, education, etc.) were collected for pregnant people. Characteristics collected for HCPs included demographic characteristics, professional experience, patient population treated, and practice setting.

### 2.4. Statistical Analyses

The data for HCPs and pregnant people were analyzed separately using IBM SPSS^®^ v28.0 (IBM Corp., Armonk, NY, USA) for descriptive statistics [24] and Sawtooth’s Lighthouse Studio^®^ v9.13.1 (Sawtooth Software, Provo, UT, USA) for DCE and BWS analyses [25]. Descriptive statistics were used to characterize the sample population for each cohort. Means and standard deviations (SDs) were reported for continuous or discrete (i.e., count) variables, whereas frequencies and percentages were reported for categorical variables.

DCE and BWS analyses were performed according to the recommendations of the ISPOR Good Research Practices for Conjoint Analysis Task Force [16,26,27,28]. To estimate preference weights for all attribute levels in the DCE and all items in the BWS, a hierarchical Bayesian (HB) model was fitted to the choice data from each exercise [13,28]. The underlying choice-probability model in the HB analysis was conditional logit, using effects coding for the attribute levels. The model assumes that the preference weights are normally distributed across respondents in the sample. This model is “hierarchical” because it has two levels. At the higher level, the model assumes that individuals’ preference weights are described by a multivariate normal distribution, and, at the lower level, the individual’s preference weights are governed by a multinomial logistic model [29].

For the DCE, mean preference weights for each sample were calculated as point estimates of the HB model coefficients, as well as standard errors (SEs) and 95% confidence intervals (CIs). Preference weights measure relative preference, which means that only changes between attribute-level estimates and the magnitude of those changes across attributes have meaningful interpretations. Based on these preference weights, potential trade-offs between utility gains and losses across attributes can be evaluated. The RI of each attribute was calculated at the respondent level by dividing the utility range of each attribute (i.e., preference weight of the most preferred level minus preference weight of the least preferred level) by the sum of the ranges of all attributes and multiplying by 100 so that the RI estimates sum to 100% across attributes. The RI estimates explain how much the range of each attribute accounts for the variation in preferences. Additional analysis of the DCE data included examination of the frequency with which the opt-out option was selected in the choice tasks out of the total number of times it was displayed.

For the BWS, HB coefficients were estimated for each respondent to capture the relative ranking assigned to each BWS attribute evaluated in the choice tasks. The estimated coefficients were transformed with logit modelling and then converted into selection probabilities and standardized to a score of 0% to 100% for reporting [13,28], with higher scores indicating a higher rank.

To evaluate potential differences in preferences among respondents, the following prespecified comparisons were performed: by cohort (HCPs vs. pregnant people), among HCPs by patient population treated (infants only vs. pregnant people only vs. both infants and pregnant people), and among pregnant people by number of births (first birth vs. second/later birth). Specifically, independent-samples t-tests were used to determine whether the preference weights and RI estimates significantly differed between a pair of subgroups. RI was compared attribute-by-attribute (a joint test was not performed). *p*-values <0.05, two-tailed, were considered statistically significant for these comparisons.

## 3. Results

In total, 314 HCPs and 1025 pregnant people completed the study survey. Quality control checks were performed on survey responses, which identified data from 4 HCPs for exclusion due to straight-lining on the DCE choice tasks (i.e., chose only preventive option A or only preventive option B for every choice task). The quality checks also identified the data from 33 pregnant people for exclusion. Of these, n = 21 were excluded for straight-lining on the DCE. Additionally, we flagged n = 6 for either providing illogical responses (e.g., rating lower preventive effectiveness as better than higher effectiveness) on >1 survey item rating the levels for each attribute (Likert scale with options from 1 = very bad to 5 = very good) or for speeding on the DCE (i.e., completing each choice task in <5 s, on average), with n = 6 flagged for having both of these violations. Upon further evaluation of their data, all 12 of these respondents had >1 illogical preference weight in the DCE (e.g., a preference weight indicating lower preventive effectiveness was preferred over higher effectiveness) and were thus excluded from analyses. Following the completion of data quality checks, the final sample sizes of 310 HCPs and 992 pregnant people were included in analyses.

### 3.1. Sample Characteristics

Table 3 reports the demographic and practice characteristics for the cohort of HCPs, and Table A1 shows these results stratified by patient population treated. Slightly more than half of HCPs were male (52.6%); most were White (71.3%) and aged ≥45 years (60.0%). At the time of the study, they had practiced medicine for an average (±SD) of 16.5 ± 8.8 years, spending a mean of 93.7 ± 9.1% of their time in direct patient care. Most HCPs reported a professional designation of Doctor of Medicine (MD) or Doctor of Osteopathic Medicine (DO) (86.1%), and 13.9% practiced as physician assistants, nurse practitioners, or midwives. Approximately 2-in-5 clinicians provided care for pregnant people but not infants (39.7%), 31.0% treated both infants and pregnant people, and 29.4% treated infants but not pregnant people. HCPs were relatively evenly distributed across the four US Census regions. Nearly half of HCPs (48.7%) reported their practice is located in either a major metropolitan or an urban area, and 30.6% reported their practice is located in the suburb of a large city.

Table 4 presents the demographic characteristics for the cohort of pregnant people, and Table A2 shows these results stratified by number of births. The mean (±SD) age of pregnant people in this study was 30.0 ± 6.3 years, and 3-in-5 respondents were pregnant for their second or later birth (60.3%). Two-thirds of pregnant people were White only (66.1%), and approximately a quarter (27.2%) self-identified as Hispanic/Latina. The largest proportion of pregnant people resided in the South (45.2%), and approximately 2-in-5 pregnant people lived in either a major metropolitan or urban area (41.4%). Nearly half of pregnant people had achieved a college degree or higher education (41.0%). At the time of the survey, a majority of pregnant people were currently employed (61.0%), and about a quarter (27.7%) reported a household income of less than $30,000. Overall, individual or family insurance plans were the most common current healthcare insurance among pregnant people (50.0%), followed by Medicaid (41.2%) and the Children’s Health Insurance Program (CHIP; 11.2%).

### 3.2. DCE Findings

**Figure 2:**
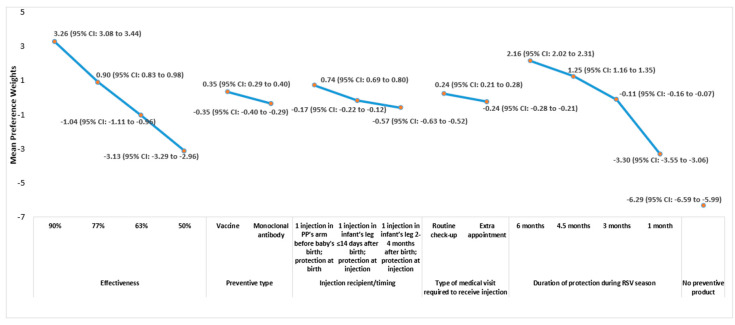
RSV Preventive Attribute-Level Mean Preference Weights in DCE: HCPs Note. Preference weights should not be interpreted by themselves. Instead, the magnitude of change within one attribute should be compared to change within another attribute. Mean preference weights are shown for each attribute-level. Attributes are shown in the order in which they were presented to respondents in the DCE choice tasks. For each attribute, the sum of preference weights equals zero. The change in preference associated with a change in the levels of each attribute is represented by the vertical distance between the preference weights for any two levels of that attribute. Larger differences between preference weights indicate that the change between those two levels is relatively more influential to overall preference. For example, increasing effectiveness from 50% to 63% yielded a change in utility of approximately 2.1 (i.e., −1.0–[−3.1]), and increasing the duration of protection from 4.5 months to 6 months yielded a change in utility of approximately 0.9 (i.e., 2.2–1.3), indicating that an incremental increase in effectiveness from 50% to 63% is 2.3 times as important to HCPs as an increase in the duration of protection from 4.5 months to 6 months (i.e., 2.1 ÷ 0.9). However, increasing the duration of protection from 1 month to 3 months yielded a change in utility of approximately 3.2 (i.e., −0.1–[−3.3]), indicating that increasing the duration of protection from 1 month to 3 months was 3.6 times as important as increasing the duration of protection from 4.5 months to 6 months (i.e., 3.2 ÷ 0.9). The large negative estimate of the “No Preventive Product” option (−6.29) indicates that HCPs are more likely to choose an RSV preventive option than the opt-out option in each choice task. Abbreviations. CI: confidence interval; HCP: healthcare provider; RSV: respiratory syncytial virus.

**Figure 3:**
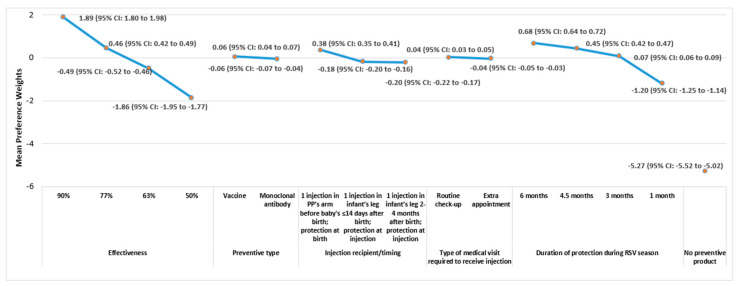
RSV Preventive Attribute-Level Mean Preference Weights in DCE: Pregnant People. Note. Preference weights should not be interpreted by themselves. Instead, the magnitude of change within one attribute should be compared to change within another attribute. Mean preference weights are shown for each attribute-level. Attributes are shown in the order in which they were presented to respondents in the DCE choice tasks. For each attribute, the sum of preference weights equals zero. The change in preference associated with a change in the levels of each attribute is represented by the vertical distance between the preference weights for any two levels of that attribute. Larger differences between preference weights indicate that the change between those two levels is relatively more influential to overall preference. For example, increasing effectiveness from 63% to 77% yielded a change in utility of approximately 0.9 (i.e., 0.4–[−0.5]), and increasing the duration of protection from 1 month to 3 months yielded a change in utility of approximately 1.3 (0.1–[−1.2]), indicating that increasing the duration of protection from 1 month to 3 months was 1.4 times as important to pregnant people as increasing product effectiveness from 63% to 77% (i.e., 1.3 ÷ 0.9). However, increasing effectiveness from 77% to 90% yielded a change in utility of approximately 1.5 (1.9–0.4), indicating that to pregnant people, increasing effectiveness from 77% to 90% was 1.7 times as important as increasing effectiveness from 63% to 77% (i.e., 1.5 ÷ 0.9). The large negative estimate of the “No Preventive Product” option (−5.27) indicates that pregnant people are more likely to choose an RSV preventive option than the opt-out option in each choice task. Abbreviations. CI: confidence interval; PP: pregnant people; RSV: respiratory syncytial virus.

Mean attribute-level preference weights from the DCE exercise are shown in Figure 2 and Figure 3 for HCPs and pregnant people, respectively. The vertical distance between the preference weights for any two levels of a given attribute (i.e., the absolute value of the difference between the preference weights for the two levels) represents the change in preference associated with a change in those two attribute levels. Larger differences between preference weights indicate that the change between those two levels is more influential to overall RSV preventive preference, relative to changes between two attribute levels associated with smaller differences in preference weights. For example, for HCPs, an increase from 3 months to 4.5 months in duration of protection during RSV season (difference between mean preference weights: 1.25–[−0.11] = 1.36) was more influential to preferences than an increase from 4.5 months to 6 months in duration of protection during RSV season (2.16–1.25 = 0.91), as 1.36 > 0.91. Similarly, an increase from 3 months to 4.5 months in duration of protection during RSV season (0.45–0.07 = 0.38) was more influential to the preferences of pregnant people than an increase from 4.5 months to 6 months in duration of protection during RSV season (0.68–0.45 = 0.23), as 0.38 > 0.23. Additionally, an increase from 3 months to 4.5 months in duration of protection during RSV season was 1.49 and 1.65 times as influential to preferences as an increase from 4.5 months to 6 months in duration of protection during RSV season for HCPs (i.e., 1.36 ÷ 0.91) and pregnant people (i.e., 0.38 ÷ 0.23), respectively.

The preference weight for the opt-out reflects the binary choice of an RSV preventive (coded as 0) or the ‘No Preventive Product’ option (coded as 1); a negative preference weight indicated a preference for the former option, and a positive preference weight indicated a preference for the latter. For HCPs and pregnant people, the absolute values of the preference weights for the ‘No Preventive Product’ option (−6.29 and −5.27, respectively) far exceeded the maximum possible differences in preference weights between levels for any of the attributes included in the DCE exercise, and the preference weights had a negative sign, which taken together, indicate a strong preference to use an RSV preventive product over the ‘No Preventive Product’ alternative (Figure 2 and Figure 3). Indeed, HCPs and pregnant people chose an RSV preventive profile over the opt-out option in 96.0% and 89.2% of choice tasks, respectively, regardless of the characteristics of the preventive options provided in the choice tasks.

The preferences of HCPs and pregnant people for preventive products were most sensitive to changes in the product’s effectiveness in preventing severe RSV illness and in the duration of protection during the RSV season (Figure 2 and Figure 3). For both HCPs and pregnant people, the preferences for increases in effectiveness and duration of protection during the RSV season appear to be ordered as expected, with preference weights increasing as the respective attribute became more favorable (i.e., higher effectiveness and longer duration of protection during the RSV season). Among both cohorts, the pattern of preference weights indicated that vaccine was preferred over mAb as the preventive type, pregnant person was preferred over infant as the injection recipient, and routine check-up was preferred over an extra appointment to receive the injection. However, changes in these three attributes had less impact on preventive choice, as indicated by the smaller differences in preference weight estimates between the levels of these attributes.

**Figure 4:**
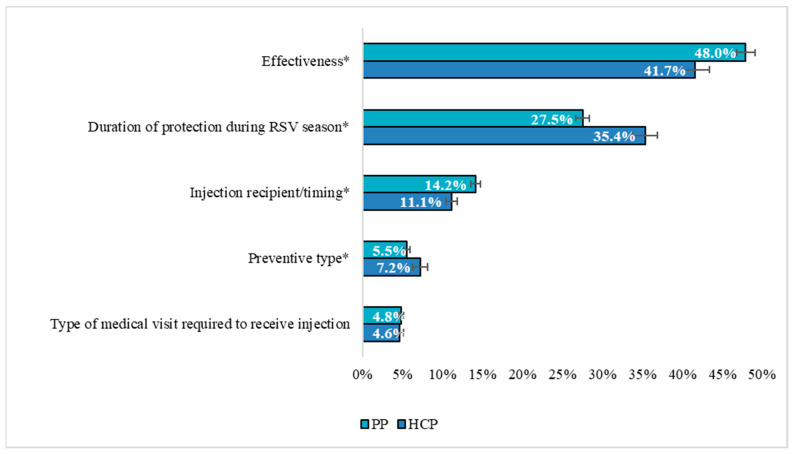
Relative Importance of RSV Preventive Attributes in DCE: HCPs vs. Pregnant People. Note. Relative importance estimates sum to 100% across attributes for each group. Error bars represent 95% confidence intervals. Attributes are presented in descending order of importance. * *p* < 0.01, 2-tailed. Abbreviations. HCP: healthcare provider; PP: pregnant people; RSV: respiratory syncytial virus.

The RI estimates for all attributes in the DCE, which reflect the proportion of variance in RSV preventive choice explained by each attribute, are shown in Figure 4. For both HCPs and pregnant people, improvement in effectiveness was the most important to preventive choice, followed by increasing the duration of protection during the RSV season and changes to the injection recipient/timing of protection, preventive type, and the type of medical visit required to receive the injection. Together, the two most important attributes accounted for approximately three-quarters of the variance in RSV preventive choice for both cohorts (HCPs: 77.1%, pregnant people: 75.5%). Nonetheless, changes to the injection recipient/timing of protection, preventive type, and the type of medical visit required to receive the injection combined to explain roughly a quarter of the variance in RSV preventive choice for HCPs and pregnant people, and each of these attributes influenced RSV preventive choice, as indicated by RI values >0% and 95% CIs that do not include 0%.

The RI of all attributes evaluated in this study differed significantly between cohorts (all, *p* < 0.01), except for the type of visit required to receive injection. Specifically, compared with HCPs, pregnant people placed greater relative importance on increased effectiveness (41.7% vs. 48.0%) and a change in injection recipient/timing of protection (11.1% vs. 14.2%). Conversely, from the perspective of HCPs, an extended duration of protection during the RSV season (35.4% vs. 27.5%) and a change in preventive type from mAb to maternal vaccine (7.2% vs. 5.5%) were more influential to RSV preventive choice. Nevertheless, despite differences in most RI estimates, the rank order of importance of preventive product attributes in the DCE was consistent across HCPs and pregnant people, suggesting the same factors drive their choice of RSV preventive.

### 3.3. BWS Findings

**Figure 5:**
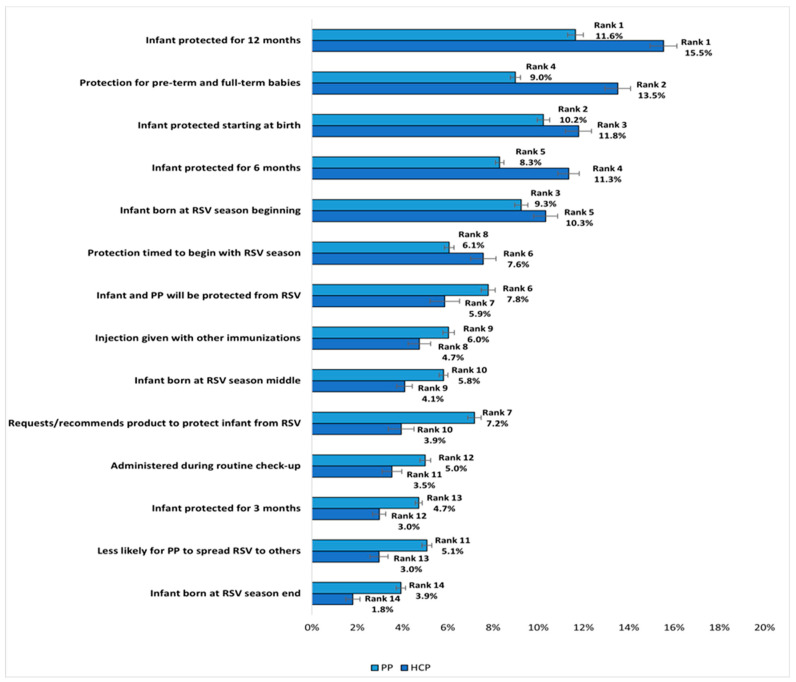
Relative Importance of RSV Preventive Attributes in BWS: HCPs vs. Pregnant People. Note. Relative importance estimates sum to 100% across attributes for each group. Error bars represent 95% confidence intervals. All comparisons between HCPs and pregnant people were statistically significant at *p* < 0.001, 2-tailed. The attributes are presented in descending order of importance estimates for HCPs. Abbreviations. HCP: healthcare provider; PP: pregnant people; RSV: respiratory syncytial virus.

The RI estimates from the BWS choice tasks, which reflect the proportion of variance in RSV preventive preference explained by each attribute, are shown in Figure 5. When evaluating a broader set of hypothetical RSV preventive product attributes in the BWS exercise, both cohorts included the same features among the top five most desirable, albeit with slightly different ranking. The most important attributes for both cohorts consisted of ‘infant protected for 12 months’, ‘infant protected for 6 months’, ‘protection for pre-term and full-term babies’, ‘infant protection starting at birth’, and ‘infant protection starting at the beginning of the RSV season’. Collectively, these five attributes accounted for the majority of variation in the RSV preventive preferences of HCPs (62.5%) and nearly half of the variation in the preferences of pregnant people (48.4%). While both HCPs and pregnant people most valued ‘infant protected for 12 months’ (both, Rank 1), ‘infant protection starting at birth’ (HCPs: Rank 3 vs. pregnant people: Rank 2) and ‘infant protection starting at the beginning of the RSV season’ (HCPs: Rank 5 vs. pregnant people: Rank 3) were slightly less important to HCPs, who placed greater priority on ‘protection for pre-term and full-term babies‘ (HCPs: Rank 2 vs. pregnant people: Rank 4), as well as on ‘infant protected for 6 months’ (HCPs: Rank 4 vs. pregnant people: Rank 5).

Relative to the other 12 attributes examined in the BWS, ‘infant protected for 3 months’ and ‘protection for an infant born at the end of the RSV season’ were the least important across both cohorts. Together, these two attributes explained <10% of the variance in RSV preventive preference for HCPs (4.8%) and pregnant people (8.3%). Nevertheless, among both cohorts, the RI estimates for these two attributes were >0%, and the 95% CIs excluded 0%, indicating that these two attributes had an influence, although relatively weak, on RSV preventive preferences. Overall, the RI assigned by HCPs showed a clear rank ordering that significantly differentiated their preferences between most of the attributes, whereas the priorities of pregnant people were more evenly distributed, with fewer significant differences between attributes. Statistically significant differences between HCPs and pregnant people were observed on all attributes in the BWS choice tasks (all, *p* < 0.001), as shown in Figure 5.

### 3.4. Subgroup Preferences among HCPs

**Figure A2:**
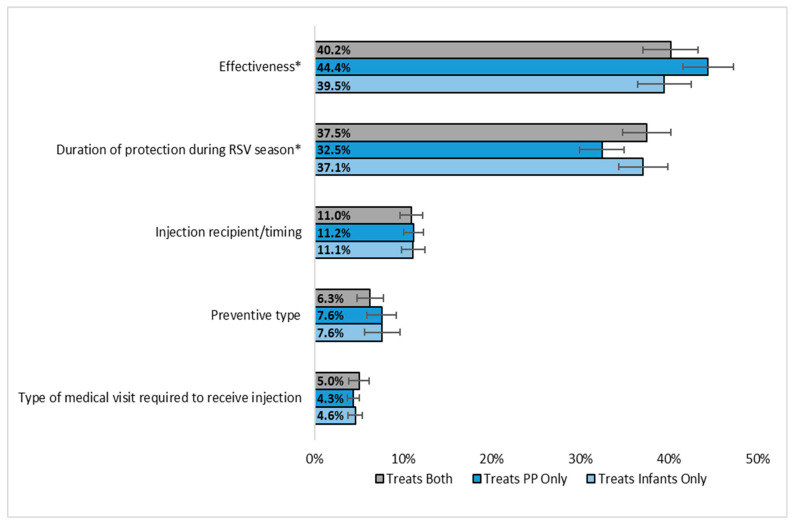
Relative Importance of RSV Preventive Attributes in DCE: HCPs by Patient Population Treated. Note. Relative importance estimates sum to 100% across attributes for each group. Error bars represent 95% confidence intervals. Attributes are presented in descending order of importance. * *p* < 0.05, 2-tailed. Abbreviations. PP: pregnant people; RSV: respiratory syncytial virus.

**Figure A3:**
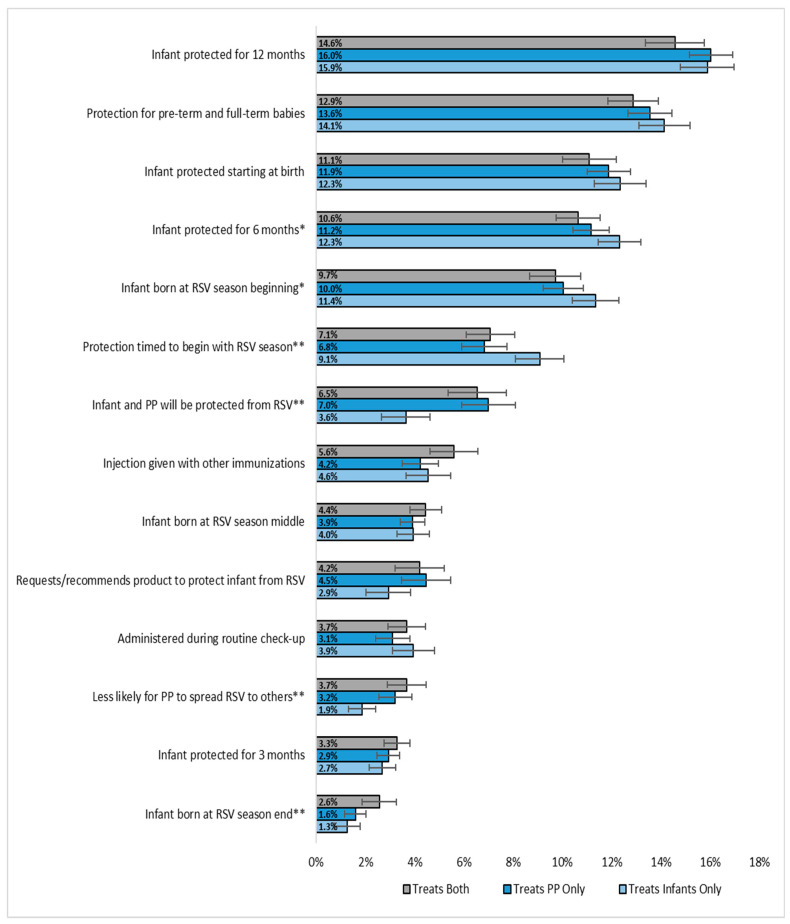
Relative Importance of RSV Preventive Attributes in BWS: HCPs by Patient Population Treated. Note. Relative importance estimates sum to 100% across attributes for each group. Error bars represent 95% confidence intervals. The attributes are presented in descending order of importance for those who treat both pregnant people and infants. * *p* < 0.05, 2-tailed; ** *p* < 0.01, 2-tailed. Abbreviations. PP: pregnant people; RSV: respiratory syncytial virus.

Generally, while some variation was observed, attributes in the DCE and BWS choice tasks were ranked similarly across HCP groups (Figure A2 and Figure A3). Significant differences in the overall distribution of RI estimates for attributes in the DCE were observed by patient population treated (*p* = 0.038), such that improvements in effectiveness were most important to HCPs who treat pregnant people but not infants (44.4%), followed by HCPs who treat both infants and pregnant people (40.2%) and those who treat infants but not pregnant people (39.5%).

Additionally, significant differences in the overall distribution of RI estimates were observed by patient population treated (*p* = 0.010), with increasing the duration of protection during the RSV season being most important to HCPs who treat both infants and pregnant people (37.5%), followed by HCPs who treat infants but not pregnant people (37.1%) and those who treat pregnant people but not infants (32.5%).

The rank order of the top five BWS attributes was the same for HCPs by patient population treated. Statistically significant differences in the overall distribution of RI estimates were observed by patient population treated on six attributes included in the BWS exercise (all, *p* < 0.05). ‘Infant protected from RSV for 6 months’, ‘infant due to be born at the beginning of the RSV season’, and ‘protection can be timed to begin with the RSV season’ were numerically more important to preventive choice for HCPs who treat infants (but not pregnant people) than for those who treat pregnant people (but not infants) or who treat both infants and pregnant people.

‘Infant and pregnant person can be protected from RSV’ and ‘injection makes it less likely the pregnant person will spread RSV to others’ were numerically more important to preventive choice for HCPs who treat pregnant people (but not infants) or who treat both infants and pregnant people, than for those who treat infants (but not pregnant people). Additionally, ‘infant due to be born at the middle of the RSV season’ was numerically more important to preventive choice for HCPs who treat both infants and pregnant people, than to those who treat infants (but not pregnant people) or who treat pregnant people (but not infants).

### 3.5. Subgroup Preferences among Pregnant People

**Figure A4:**
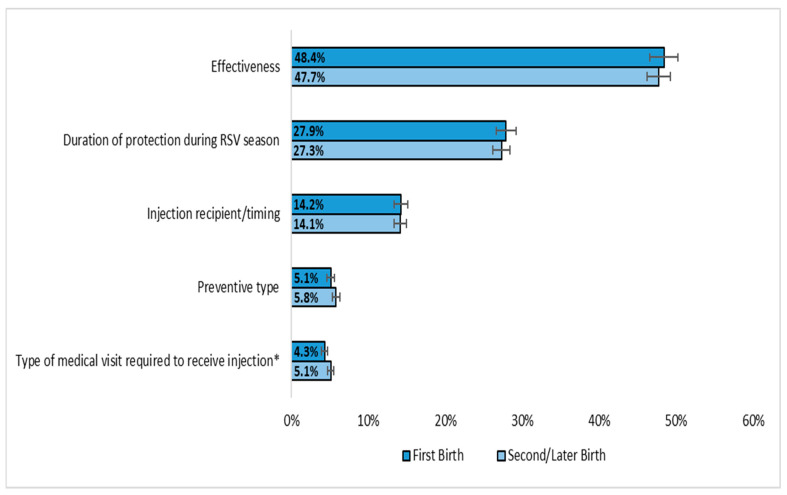
Relative Importance of RSV Preventive Attributes in DCE: Pregnant People by Number of Births. Note. Relative importance estimates sum to 100% across attributes for each group. Error bars represent 95% confidence intervals. Attributes are presented in descending order of importance. * *p* < 0.05, 2-tailed. Abbreviations. RSV: respiratory syncytial virus.

**Figure A5:**
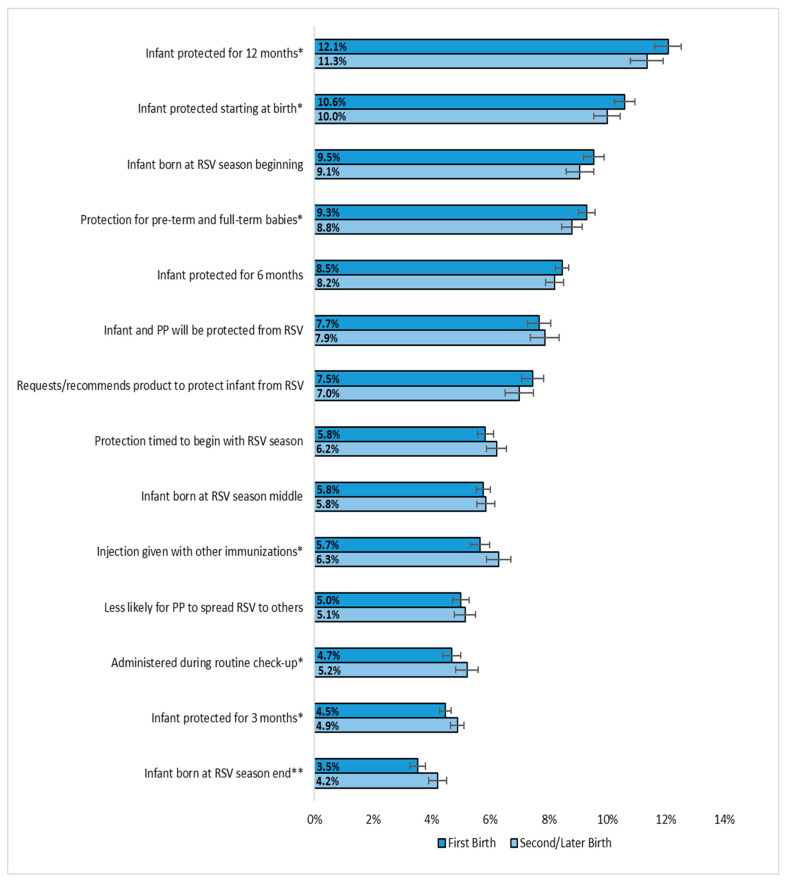
Relative Importance of RSV Preventive Attributes in BWS: Pregnant People by Number of Births. Note. Relative importance estimates sum to 100% across attributes for each group. Error bars represent 95% confidence intervals. The attributes are presented in descending order of importance for those expecting their first birth. * *p* < 0.05, 2-tailed; ** *p* < 0.01, 2-tailed. Abbreviations. RSV: respiratory syncytial virus.

The rank order of the RI estimates from the DCE and BWS choice tasks was mostly consistent among pregnant people by number of births; however, modest variation in the rank order of BWS attributes was observed between these subgroups (Figure A4 and Figure A5). Additionally, in the DCE, change in the type of visit required to receive the injection was more important to pregnant people on their second/later birth than to those on their first birth (RI = 5.1% vs. 4.4%, *p* = 0.014), although this difference was small in magnitude, and this was the least important attribute for both groups (Figure A4).

Statistically significant differences (all, *p* < 0.05) between pregnant people by number of births were observed on some of the BWS attributes. For instance, ‘infant protected from RSV for 12 months’, ‘infant protected from RSV at birth’, and ‘provides protection from RSV for pre-term and full-term infants’ were more important to preventive choice for pregnant people having their first birth than for those having their second/later birth. ‘Injection can be administered with other immunizations’, ‘injection can be administered during a routine check-up’, ‘infant protected from RSV for 3 months’, and ‘infant due to be born at the end of RSV season’ were more important to preventive choice for pregnant people having their second/later birth than for those having their first birth.

## 4. Discussion

Within the broadening landscape of preventive products targeting infant protection from serious RSV illness, both HCPs and pregnant people may encounter complex choices. To our knowledge, this research is the first to quantify preferences for the most salient factors regarding preventive products to protect infants against serious illness from RSV. Notably, both HCPs and pregnant people overwhelmingly chose an RSV preventive over the opt-out option (96.0% and 89.2% of times, respectively). Indeed, participants in both cohorts assigned, on average, a high negative utility to the ‘No Preventive Product’ alternative, indicating a strong receptivity to using an intervention to protect infants from serious illness due to RSV.

When evaluating each feature of a hypothetical preventive product to protect infants from serious RSV illness, the priorities of respondents in both cohorts aligned, overall. HCPs and pregnant people most valued improvement in effectiveness from 50% to 90%, followed by an increase in duration of protection during RSV season from 1 month to 6 months. While extending the duration of protection during the high-risk season was a key driver of preferences, DCE results showed that it may be offset by efficacy gains. For example, improvement in the effectiveness of the preventive product was 1.2 times as important to HCPs and 1.7 times as important to pregnant people as greater duration of protection during RSV season.

While palivizumab is indicated to prevent RSV in high-risk infants and young children, it requires multiple doses and is costly. Hence, the recently approved maternal vaccine and long-acting mAbs can offer more convenient options for RSV prophylaxis and are not limited to high-risk populations; furthermore, both of these new preventive options are projected to be cost-effective [30]. Of importance, cooperation between prenatal and pediatric HCPs, as well as relevant governmental and non-governmental agencies, will be needed to facilitate broad access to these preventives in order to realize the potential public health benefits of RSV immunization [30]. Understanding the preferences and priorities of key stakeholders, such as pregnant people and HCPs, may help inform efforts to spur greater immunization coverage, which can, in turn, reduce the substantial mortality and morbidity burden associated with severe illness from RSV among infants.

Notably, prior studies have found that <70% of pregnant people intend to receive Tdap, influenza, or COVID-19 vaccination during their pregnancy or to have their infant immunized [31,32], suggesting suboptimal immunization coverage for vaccine-preventable infections. Given the importance of protecting infants from severe RSV illness, it is necessary to understand and overcome potential barriers to immunization. In the US, the CDC specifically recommends that the RSV vaccine be co-administered with other recommended vaccines, including Tdap, influenza, and COVID-19, during pregnancy [33]. As the ‘No Preventive Product’ option was infrequently selected by pregnant people in the current study, it is possible that those who preferred a vaccine over a long-acting mAb to protect their infant against serious illness from RSV may also be more willing to receive other recommended vaccines during their pregnancy that can confer infant immunity. Future research will need to confirm whether the use of a maternal RSV vaccine will improve coverage for Tdap, influenza, and/or COVID-19 vaccination through co-administration.

Compared with available national data [34,35], HCPs in this study were less often male (54% vs. 62%) but more frequently White (71% vs. 56%). However, we observed similar proportions of HCPs in the current study and in the national data who were African American or Black (3% vs. 5%), Asian (each, 17%), Hispanic or Latino (8% vs. 6%), and older than age 45 years (60% vs. 64%) [34,35]. Any differences between the national data and HCPs in the current study are likely because the former reflects the demographic characteristics of US physicians (MD and DO) across all specialties, whereas this study included HCPs (physicians, nurse practitioners, and physician assistants) who treat pregnant people and/or infants (e.g., OB/Gyn and pediatrics). The sociodemographic characteristics and the distribution of pregnant people by number of births aligned with the statistics reported on live births by the CDC [36,37]. For example, the average age of pregnant people in the current study was similar to the mean of all individuals with live births in 2020 (mean ± SD: 30.0 ± 6.3 vs. 29.2 ± 5.8 years), with comparable proportions being pregnant for the first time (40% vs. 38%), and somewhat higher rates of college graduates (41% vs. 34%) and White participants (70% vs. 51%). Compared with national data from the US Census Bureau [38], pregnant people in this study tended to be somewhat overrepresented among those with the lowest annual household income (<$15,000: 14% vs. 7%) and underrepresented among those with the highest annual household income (≥$150,000: 8% vs. 18%). Thus, the samples of HCPs and pregnant people in the current study were generally representative of their respective broader populations in the US, enhancing the generalizability of findings.

### 4.1. Results Interpretation: Overall Cohorts of Pregnant People and HCPs

A systematic review of choice experiments reported that vaccine effectiveness was the most preferred attribute for both vaccinees and their guardians [39], which aligns with the findings of this study. A global study eliciting attitudes about RSV immunization in infants found that, for parents, safety and efficacy were the most important immunization attributes [40]. A prior DCE study eliciting preferences for a maternal Group B Streptococcus vaccine among pregnant people in the US and Ireland also reported that vaccine efficacy and protecting the unborn infant from serious infection were the most important attributes [41]. Results from the current study, which highlight the high importance of efficacy for pregnant people, are consistent with these observations. Furthermore, the preferences of HCPs were likewise most strongly driven by RSV preventive effectiveness. Notably, for both cohorts, increased effectiveness and duration of protection during RSV season jointly explained over three-quarters of the variation in RSV preventive choice. These findings suggest that these two factors should be the primary emphasis of public health educational initiatives to inform pregnant people and HCPs about the benefits of immunization against RSV.

Perhaps less apparent, the preference for a maternal vaccine across cohorts was driven in part by the recipient of injection, which ranked as the third most influential attribute in the DCE, rather than the preventive type, which was the fourth most important attribute. Relative to the importance of having a vaccine instead of a mAb, having a maternal injection instead of an infant injection was 1.5 times as important to HCPs but 2.6 times as important to pregnant people. These findings also advance the supposition that pregnant people are more concerned with the newborn’s safety during the first months of life and align with prior qualitative research [42,43]. Prior research has highlighted vaccine hesitancy among HCPs in Western countries, which can, in turn, contribute to reduced vaccine uptake among patients [44]. While the current study’s results suggest that, on average, a maternal vaccine was preferred over a mAb by both HCPs and pregnant people, further research will be needed to determine whether and to what extent vaccine hesitancy influences RSV preventive preferences.

Despite the alignment between HCPs and pregnant people when ranking features of RSV preventive products, the RI of all attributes evaluated in this study varied significantly between cohorts, except for the type of visit required to receive the injection. For instance, when selecting a preventive, pregnant people were more likely to consider efficacy gains and the injection recipient, whereas HCPs were more influenced by an extended duration of protection during the RSV season and by the type of prevention. Since evaluating the differences between a vaccine and a mAb requires a certain degree of medical literacy, it is not surprising that the type of prevention was of greater relative importance to HCPs than to pregnant people.

Consistent with the DCE results, data from the BWS exercise showed that both HCPs and pregnant people preferred a longer duration of protection during the RSV season, and having the infant protected from RSV starting at birth was highly important. Similar to the DCE results, the BWS also showed that the type of visit required to receive the injection had relatively less importance in RSV preventive product choice. Thus, both preference elicitation methodologies generally yielded a similar pattern of findings for HCPs and pregnant people. Results from the DCE and BWS exercises suggest that HCPs and pregnant people are aligned in their perceptions of the most and least preferred features of RSV preventives. These insights may, in turn, help to facilitate shared decision-making regarding RSV immunization options. Nonetheless, the set of attributes presented in the DCE and BWS choice tasks were not identical (e.g., effectiveness and preventive type were evaluated in the DCE but not included in the BWS), thereby precluding direct comparisons between results observed with DCE versus those observed with BWS.

### 4.2. Results Interpretation: Subgroups

In subgroup analyses, HCPs who treat pregnant people (but not infants) were significantly more likely to value improvement in effectiveness, and less likely to value a prolonged duration of protection during the RSV season, compared with other provider groups. Even though maternal vaccination is a safe and effective means of immunizing pregnant people and their infants against a number of preventable infections, guidelines on what timing or dosage is appropriate for different vaccines remain elusive [17]. Maternal vaccination is inherently associated with uncertainty around the time required to build maternal antibodies, variability in response, and the possibility of a premature delivery. Consequently, for HCPs who exclusively manage pregnant people, increasing the vaccine’s efficacy maximizes the chance of successfully protecting the newborn after birth, which may or may not occur during the RSV season.

When evaluating BWS attributes, HCPs who exclusively treat infants viewed ‘infant protection from RSV illness for 6 months’, ‘protection for infant due to be born at beginning of RSV season’, and the ability to ‘time protection at the beginning of RSV season’ as high in importance and placed less importance on ‘protection from RSV for both infant and pregnant person’ and the ‘reduced risk for the pregnant person to spread RSV to others’. These observations highlight a general alignment among HCPs regarding perceived benefits of preventive interventions. Conceivably, HCPs caring exclusively for infants target preventive characteristics that maximize the safety of this patient population, while HCPs in other groups may have a broader perspective on preventive interventions.

The BWS results indicate that pregnant people expecting their first birth were more concerned with infant protection starting at birth and lasting for 12 months, as well as with the ability to protect both preterm and full-term infants, whereas those who have experienced previous births placed higher value on the modality of preventive administration and convenience. It is not surprising that, for pregnant people expecting their first birth, infant safety is paramount; therefore, minimizing the risk of infection is the most important goal that offsets other potential benefits. On the other hand, it may be reasonable to assume that individuals who have experienced previous births may have greater involvement in caring for children; thus, they are more inclined to evaluate the modality of the preventive intervention, seeking potential gains in time, effort, and convenience. While it is possible that differences in RSV awareness could have influenced the preferences of pregnant people, preferences were generally similar (including rank order of attributes) by number of prior births. Moreover, the few differences observed between subgroups of pregnant people were small in magnitude. Future studies should examine the degree to which continued differentiation is necessary to better tailor communication regarding RSV preventives.

### 4.3. Strengths and Limitations

There were several strengths and limitations of the current study that should be considered when interpreting the results. First, as inherent with any research relying on convenience sampling methods, it is possible that certain subgroups of HCPs and pregnant people may be overrepresented, and the results may not be generalizable to the entire population. Nonetheless, as previously described, the demographic characteristics of the study samples were generally representative of the broader population of US HCPs and pregnant people [34,35,36,37,38], which is a strength of this study. Survey response rates, which could not be determined for the current study (the total number of invitations sent by the panels was not disclosed to the researchers), may also impact the representativeness of the study samples. While a prior review reported response rates of up to 68% for survey research methods [45], a recent meta-analysis found that online surveys have lower response rates than alternative data collection methods [46].

Second, the self-reported nature of the survey data is associated with potential corresponding biases such as inaccurate recall and false reporting (whether intentional or unintentional). For example, the number of births reported by pregnant people was not confirmed by medical records. However, this limitation was expected to have minimal impact on the results, as the primary endpoints of this study, RSV preventive preferences and choice, were a function of subjective respondent perceptions.

Third, when asking HCPs about their professional designation, MD and DO were included as a single response option in the survey, which precluded the ability to analyze these physicians separately. Due to differences in medical training between MD and DO physicians, we cannot exclude the possibility that preferences may vary by professional designation. Nonetheless, in the US, MD and DO physicians generally perform similar functions in clinical practice, and the study eligibility criteria required that they all had recent experience (past 6 months) prescribing, recommending, and/or administering immunizations for pregnant people and/or infants.

Fourth, stated preferences may not perfectly correspond to actual RSV preventive choices. However, a prior meta-analysis found that DCEs are able to reasonably approximate individuals’ actual healthcare choices [47], providing greater confidence in the inferences drawn from the current study’s findings. The DCE and BWS in this study were designed to closely reflect clinical evidence regarding available preventives, as well as maternal vaccines and mAbs that were recently approved or currently in development, which is a strength of the study, although the DCE and BWS cannot capture all aspects involved in real-world immunization decisions. Furthermore, unlike DCE, BWS attributes may not reflect the characteristics of currently available preventive choices (e.g., the maximum duration of protection of currently available preventive products is 6 months; hence, the preference for 12 months of protection has informative value but is not relevant in clinical practice).

Lastly, this study was limited to US-based HCPs and pregnant people. Accordingly, the results may not reflect the views of pregnant persons or HCPs outside of the US. Future research will be needed to confirm the extent to which the findings generalize beyond the US healthcare context.

## 5. Conclusions

Taken together, the findings demonstrate agreement between HCPs and pregnant people on the attributes that are most and least important to RSV preventive choice. Both HCPs and pregnant people were receptive to hypothetical RSV preventive products, with few choosing to opt-out of preventive use. Across cohorts, decisions were made primarily on product effectiveness and duration of protection; additionally, a vaccine was preferred over mAb, maternal injection was preferred to infant injection, and both cohorts preferred that the infant be protected from RSV starting at birth. However, when selecting an RSV preventive product, pregnant people were more likely to consider efficacy gains and the injection recipient, whereas HCPs were more influenced by a longer duration of protection during the RSV season and by the type of preventive product. Understanding the key determinants of immunization decision-making may help healthcare policymakers design and implement successful strategies for RSV prevention and may facilitate public health communication efforts.

## Figures and Tables

**Table 2:** Best–Worst Scaling Attributes.

Attribute	Description
Infant protected starting at birth	The baby is protected against illness caused by RSV starting at birth
Protection timed to begin with RSV season	Protection from RSV can be timed to begin with the RSV season
Infant born at RSV season beginning	The baby is due to be born at the beginning of the RSV season
Infant born at RSV season middle	The baby is due to be born in the middle of the RSV season
Infant born at RSV season end	The baby is due to be born at the end of the RSV season
Infant and pregnant person will be protected from RSV	In addition to the baby, the (pregnant woman/pregnant person) * could also be protected against illness caused by RSV
Less likely for pregnant person to spread RSV to others	Receiving the injection would make it less likely for the (pregnant woman/pregnant person) * to spread RSV to others that they come into close contact with
Injection given with other immunizations	The injection can be given along with other immunizations
Administered during routine check-up	The injection can be administered during a routine check-up appointment
Requests/recommends product to protect infant from RSV	Your healthcare provider recommends that a pregnant woman or her baby receive an injection to protect her child against RSV infection **
Infant protected for 3 months	The baby is protected against illness caused by RSV for 3 months
Infant protected for 6 months	The baby is protected against illness caused by RSV for 6 months
Infant protected for 12 months	The baby is protected against illness caused by RSV for 12 months
Protection for pre-term and full-term babies	The injection is expected to provide protection against serious illness caused by RSV for both pre-term and full-term babies

* “Pregnant woman” was displayed in the BWS for pregnant people, and “pregnant person” was displayed in the HCP version. ** The description shown for this attribute is from the BWS for pregnant people. This attribute was worded as follows in the BWS for HCPs: “Your patient requests a product to protect their baby against RSV illness.” Abbreviations. HCP: healthcare provider; PP: pregnant people; RSV: respiratory syncytial virus.

**Table 3:** Sample Characteristics: HCPs.

Variables		Total Sample (N = 310)	
Gender, % (n)	Male	52.6	163
	Female	44.5	138
	Prefer not to answer	2.9	9
Age, % (n)	<25 years	0.0	0
	25–34 years	16.1	50
	35–44 years	23.9	74
	45–54 years	27.1	84
	55–64 years	26.8	83
	65–74 years	6.1	19
	75–84 years	0.0	0
	≥85 years	0.0	0
Race, % (n) *	African American/Black	3.2	10
	Asian	17.1	53
	American Indian/Alaska Native/Native Hawaiian/Other Pacific Islander	1.3	4
	White	71.3	221
	Another race	4.2	13
	Prefer not to answer	5.2	16
Ethnicity, % (n)	Hispanic/Latino	7.7	24
Primary medical specialty, % (n)	Pediatrics	33.5	104
	Obstetrics/Gynecology	33.5	104
	Family Medicine/General Medicine	32.6	101
	Midwifery	0.3	1
Professional designation, % (n)	MD or DO	86.1	267
	PA/NP/Nurse Midwife	13.9	43
Patient population treated, % (n)	Treats infants only	29.4	91
	Treats pregnant people only	39.7	123
	Treats both infants and pregnant people	31.0	96
US region of practice, % (n)	Northeast	21.9	68
	Midwest	24.8	77
	South	32.3	100
	West	21.0	65
Practice location, % (n)	Major metropolitan area	26.1	81
	Urban area	22.6	70
	Suburb of a large city	30.6	95
	Small city	13.9	43
	Rural or small town	6.8	21
Vaccines recommended to patients during pregnancy (if treats pregnant people), % (n)	Influenza	96.8	212
	Tdap	95.4	209
	COVID-19	88.1	193
	Other vaccines	28.3	62
	None of these	0.5	1
Vaccines reccommended for infants to receive during first 6 months of life (if treats infants), % (n)	Hepatitis B	92.5	173
	Rotavirus	86.6	162
	Tdap	95.7	179
	Haemophilus influenzae Type b	92.0	172
	Pneumococcal conjugate	86.1	161
	Inactivated poliovirus	90.9	170
	Other vaccines	22.5	42
	None of these	0.5	1
Number of years in practice, mean (SD)		16.5	8.8
Percent of time spent in direct patient care, mean (SD)		93.7	9.1

* HCPs could select >1 response option. Abbreviations. COVID-19: coronavirus disease 2019; DO: Doctor of Osteopathic Medicine; MD: Doctor of Medicine; PA: physician assistant; NP: nurse practitioner; SD: standard deviation; Tdap: tetanus, diphtheria, and pertussis; US: United States.

**Table 4:** Sample Characteristics: Pregnant People.

Variables		Total Sample (N = 992)	
Age (years), mean (SD)		30.0	6.3
Race, % (n) *	White only	66.1	656
	Any other race only	28.3	281
	Multiracial	4.5	45
	Prefer not to answer	1.0	10
Ethnicity, % (n)	Hispanic/Latina	27.2	270
Current health insurance, % (n) *	Individual/family insurance plan	50.0	496
	CHIP	11.2	111
	Medicaid	41.2	409
	Other health insurance	6.0	60
	Not sure of health insurance	3.5	35
	Do not have health insurance	2.3	23
	Prefer not to answer	2.6	26
Employment, % (n)	Employed (full-time or part-time)	61.0	605
Education, % (n)	College degree or higher	41.0	407
Household income, % (n)	≤$30 k	27.7	275
	>$30 k to ≤$60 k	22.7	225
	>$60 k to ≤$90 k	20.1	199
	>$90 k	24.9	247
	Prefer not to answer	4.6	46
Number of births, % (n)	Pregnant for first birth	39.7	394
	Pregnant for second or later birth	60.3	598
US region of residence, % (n)	Northeast	14.3	142
	Midwest	20.3	201
	South	45.2	448
	West	20.3	201
Residence location, % (n)	Major metropolitan area	16.6	165
	Urban area	24.8	246
	Suburb of a large city	27.4	272
	Small city	15.1	150
	Rural or small town	16.0	159
Received influenza vaccine during this pregnancy, % (n)	Yes	47.0	466
	No	51.5	511
	Not sure	1.5	15
Received Tdap vaccine during this pregnancy, % (n)	Yes	41.1	408
	No	54.8	544
	Not sure	4.0	40
Received another vaccine during this pregnancy, % (n)	Yes	25.7	255
	No	68.1	676
	Not sure	6.1	61

* Pregnant people could select ≥1 response option. Abbreviations. CHIP: Children’s Health Insurance Program; SD: standard deviation; Tdap: tetanus, diphtheria, and pertussis.

## Data Availability

The study data are not publicly available due to the data collection only being granted exemption determination from an IRB for this specific protocol. The data presented in this study are available upon reasonable request from the corresponding author for non-commercial use.

## Appendix A

**Table A1:** Sample Characteristics: HCPs by Patient Population Treated.

Variables		Infants Only (N = 91)	PP Only (N = 123)	Both (N = 96)
Gender, % (n)	Male	56.0 (51)	48.0 (59)	55.2 (53)
	Female	42.9 (39)	49.6 (61)	39.6 (38)
	Prefer not to answer	1.1 (1)	2.4 (3)	5.2 (5)
Age, % (n)	<45 years	45.1 (41)	35.8 (44)	40.6 (39)
	≥45 years	54.9 (50)	64.2 (79)	59.4 (57)
Race, % (n) *	African American/Black	3.3 (3)	3.3 (4)	3.1 (3)
	Asian	17.6 (16)	20.3 (25)	12.5 (12)
	American Indian/Alaska Native/Native Hawaiian/Other Pacific Islander	2.2 (2)	0.0 (0)	2.0 (2)
	White	74.7 (68)	68.3 (84)	71.9 (69)
	Another race	0.0 (0)	4.9 (6)	7.3 (7)
	Prefer not to answer	3.3 (3)	4.1 (5)	8.3 (8)
Ethnicity, % (n)	Hispanic/Latino	7.7 (7)	5.7 (7)	10.4 (10)
Primary medical specialty, % (n)	Pediatrics	93.4 (85)	0.0 (0)	19.8 (19)
	Obstetrics/Gynecology	0.0 (0)	74.8 (92)	12.5 (12)
	Family Medicine/General Medicine	6.6 (6)	24.4 (30)	67.7 (65)
	Midwifery	0.0 (0)	0.8 (1)	0.0 (0)
Professional designation, % (n)	MD or DO	94.5 (86)	87.8 (108)	76.0 (73)
	PA/NP/Nurse Midwife	5.5 (5)	12.2 (15)	24.0 (23)
US region of practice, % (n)	Northeast	28.6 (26)	20.3 (25)	17.7 (17)
	Midwest	25.3 (23)	24.4 (30)	25.0 (24)
	South	27.5 (25)	34.1 (42)	34.4 (33)
	West	18.7 (17)	21.1 (26)	22.9 (22)
Practice location, % (n)	Major metropolitan area	25.3 (23)	30.9 (38)	20.8 (20)
	Urban area	27.5 (25)	18.7 (23)	22.9 (22)
	Suburb of a large city	31.9 (29)	30.1 (37)	30.2 (29)
	Small city	13.2 (12)	12.2 (15)	16.7 (16)
	Rural or small town	2.2 (2)	8.1 (10)	9.4 (9)
Number of years in practice, mean (SD)		15.5 (9.6)	17.8 (8.9)	15.9 (7.8)
Percent of time spent in direct patient care, mean (SD)		93.1 (9.7)	93.8 (7.9)	94.2 (10.1)

* HCPs could select >1 response option. Abbreviations. DO: Doctor of Osteopathic Medicine; MD: Doctor of Medicine; PA: physician assistant; PP: pregnant people; NP: nurse practitioner; SD: standard deviation; U.S.: United States.

**Table A2:** Sample Characteristics: Pregnant People by Number of Births.

Variables		First Birth (N = 394)	Second/Later Birth (N = 598)
Age (years), mean (SD)		27.7 (6.0)	31.5 (6.2)
Race, % (n) *	White only	70.6 (278)	63.2 (378)
	Any other race only	25.6 (101)	30.1 (180)
	Multiracial	3.0 (12)	5.5 (33)
	Prefer not to answer	0.8 (3)	1.2 (7)
Ethnicity, % (n)	Hispanic/Latina	26.1 (103)	27.9 (167)
Current health insurance, % (n) *	Individual/family insurance plan	55.1 (217)	46.7 (279)
	CHIP	7.1 (28)	13.9 (83)
	Medicaid	31.5 (124)	47.7 (285)
	Other health insurance	5.8 (23)	6.2 (37)
	Not sure of health insurance	3.8 (15)	3.3 (20)
	Do not have health insurance	3.6 (14)	1.5 (9)
	Prefer not to answer	2.3 (9)	2.8 (17)
Employment, % (n)	Employed (full-time or part-time)	65.7 (259)	57.9 (346)
Education, % (n)	College degree or higher	41.1 (162)	41.0 (245)
Household income, % (n)	≤$30 k	28.7 (113)	27.1 (162)
	>$30 k to ≤$60 k	23.4 (92)	22.2 (133)
	>$60 k to ≤$90 k	17.5 (69)	21.7 (130)
	>$90 k	24.4 (96)	25.3 (151)
	Prefer not to answer	6.1 (24)	3.7 (22)
US region of residence, % (n)	Northeast	16.0 (63)	13.2 (79)
	Midwest	22.3 (88)	18.9 (113)
	South	42.4 (167)	47.0 (281)
	West	19.3 (76)	20.9 (125)
Residence location, % (n)	Major metropolitan area	17.3 (68)	16.2 (97)
	Urban area	23.9 (94)	25.4 (152)
	Suburb of a large city	28.9 (114)	26.4 (158)
	Small city	15.7 (62)	14.7 (88)
	Rural or small town	14.2 (56)	17.2 (103)

* Pregnant people could select ≥1 response option. Abbreviations. CHIP: Children’s Health Insurance Program; SD: standard deviation.
